# Experimental Evolution of Resistance to Artemisinin Combination Therapy Results in Amplification of the *mdr1* Gene in a Rodent Malaria Parasite

**DOI:** 10.1371/journal.pone.0011593

**Published:** 2010-07-15

**Authors:** Louise A. Rodrigues, Gisela Henriques, Sofia T. Borges, Paul Hunt, Cecília P. Sanchez, Axel Martinelli, Pedro Cravo

**Affiliations:** 1 UEI Biologia Molecular, UEI Malária, Centro de Malária e Outras Doenças Tropicais/IHMT/UNL, Lisbon, Portugal; 2 Centre for Immunity, Infection and Evolution, School of Biological Science, The University of Edinburgh, Edinburgh, United Kingdom; 3 Abteilung Parasitologie, Hygiene Institut, Universitätsklinikum Heidelberg, Heidelberg, Germany; BMSI-A*STAR, Singapore

## Abstract

**Background:**

Lacking suitable alternatives, the control of malaria increasingly depends upon Artemisinin Combination Treatments (ACT): resistance to these drugs would therefore be disastrous. For ACTs, the biology of resistance to the individual components has been investigated, but experimentally induced resistance to component drugs in combination has not been generated.

**Methodology/Principal Findings:**

We have used the rodent malaria parasite *Plasmodium chabaudi* to select *in vivo* resistance to the artesunate (ATN) + mefloquine (MF) version of ACT, through prolonged exposure of parasites to both drugs over many generations. The selection procedure was carried out over twenty-seven consecutive sub-inoculations under increasing ATN + MF doses, after which a genetically stable resistant parasite, AS-ATNMF1, was cloned. AS-ATNMF1 showed increased resistance to ATN + MF treatment and to artesunate or mefloquine administered separately. Investigation of candidate genes revealed an *mdr1* duplication in the resistant parasites and increased levels of *mdr1* transcripts and protein. There were no point mutations in the *atpase6* or *ubp1*genes.

**Conclusion:**

Resistance to ACTs may evolve even when the two drugs within the combination are taken simultaneously and amplification of the *mdr1* gene may contribute to this phenotype. However, we propose that other gene(s), as yet unidentified, are likely to be involved.

## Introduction

Anti-malarial drug resistance poses a serious challenge for the effective control of malaria. *Plasmodium falciparum*, the causative agent of the most severe form of the disease to humans, has evolved extensive resistance to almost all known drugs, except for Artemisinin (ART) derivatives [1]. ART derivatives are currently used in combination with a chemically unrelated drug in most malaria endemic countries with the purpose of reducing the probability of drug resistance evolution [2] and possibly of augmenting therapeutic efficacies by exploiting synergistic effects of their component drugs.

The ability of Artemisinin Combination Therapy (ACT) to delay resistance evolution may depend upon whether the two drugs share the same resistance mechanisms and whether there is pre-existing resistance to one or other of the drug components. Additionally, the possibility of resistance increases if incomplete treatment is received by the patient (counterfeit drugs, non-compliance, mal-absorption, vomiting, etc) [3] or if under inadequate supervision, ACT components are used alone or at sub-optimal concentrations or intervals [4]. These scenarios allow parasites to be exposed to sub-therapeutic levels of the drug, thus contributing to the selection of tolerant strains that would later give rise to resistance.

Regions of low endemicity have historically been the cradle of newly emergent resistant parasites. For example, mefloquine- (MF) resistant *P. falciparum* parasites arose in South-East Asia after ten years of MF monotherapy [5]. In order to face increasing levels of MF resistance, the ACT version of artesunate-mefloquine (ATN + MF) was introduced in SE Asia in 1994, restoring the favourable clinical outcome with cure rates reaching as high as 100% [6]. However, recent reports of increased *in vitro* IC_50_ and reduced parasite clearance rates have raised the prospect that the efficacy of the ATN component may be fading [7]–[9]. Consequently, an understanding of the biology and genetics of ACT resistance will provide evidence-based information to monitor, delay or prevent the evolution of resistance.

The genetics of resistance to individual components of ACT in *P. falciparum* has been well-studied, but is still incompletely understood. For ART, its anti-malarial effect has been proposed to be mediated by its inhibition of the SERCA type Ca^2+^-pump, ATPase6, [10]–[11]. Mutations in the *atpase6* gene were associated with increases in artemether *in vitro* IC_50_ in South America [12], but direct experimental evidence for *atpase6* involvement has yet to be presented. Also, in two recent independent reports, the *atpase6* gene was not mutated in parasites confirmed to have markedly reduced *in vivo* susceptibility to artesunate in Western Cambodia [7]–[8].

The role of *pfmdr1* has also been extensively investigated. It encodes a P-glycoprotein whose over-expression is responsible for multi-drug resistance in cancer cells [13]. In *P. falciparum*, field studies [14]–[19], drug selection experiments [20]–[21], genetic manipulation [22], and the identification of selective sweeps around the *mdr1* locus [23]–[24], have provided evidence to support the role of *pfmdr1* gene amplification in resistance to MF and lumefantrine as well as to the chemically unrelated ART derivatives. However, as with *atpase6*, no association was found between *mdr1* genotypes and the first confirmed cases of reduced *in vivo* susceptibility to ATN [7]–[8].

**Figure 1:**
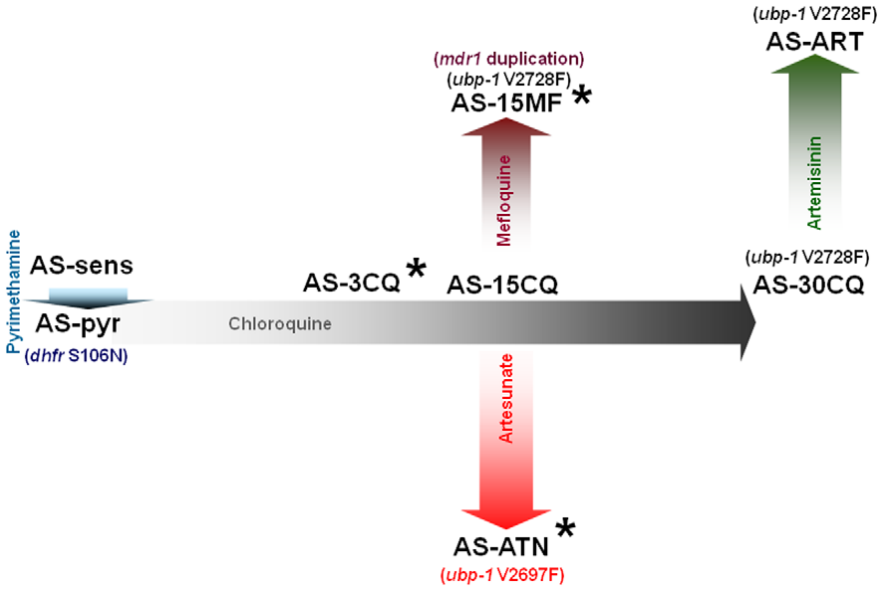
Drug resistance lineage of *Plasmodium chabaudi* AS. Note: the Figure does not depict all drug-resistant clones within the AS lineage. Only those relevant to the present work are represented. Each drug used to select resistant clones is noted inside each arrow and the increasing colour tonality of the arrow represents an approximation of the increase in drug doses during the evolution of resistance. The length of each arrow depicts the approximate relative time for generating the resistance phenotype. The generation of pyrimethamine resistance was a result of a single-step selection, whilst for all other drugs the evolution of resistance resulted from prolonged exposures to small increments in drug concentrations over many generations. The genotypic differences between a particular parasite and its progenitor are noted in brackets. Asterisks depict the clones used in this work.

The AS lineage of multidrug-resistant mutants of *Plasmodium chabaudi* (Figure 1) has been developed by experimental evolution and is a useful model to investigate questions regarding the *in vivo* biology of antimalarial resistance [25]. Because all parasites within this lineage are isogenic, direct comparisons between resistant mutants and their sensitive progenitors offers a simple and informative means of identifying mutations conferring resistance. *P. chabaudi* parasites show similar mechanisms for resistance to pyrimethamine and MF: mutations in *dhfr* [26] (in AS-PYR) and amplification of *mdr1* [27] (in AS-15MF), respectively (Figure 1).

Genetic and candidate gene analysis of an artemisinin-resistant strain of *P. chabaudi* [28] identified the locus (chromosome 2) conferring *in vivo* ART resistance and revealed two point mutations in a gene (*ubp1*) encoding a de-ubiquitinating enzyme [29]. Interestingly, a UBP-1 V2728F mutation was detected in the MF-resistant parasites AS-15MF, which had never been exposed to ART in their selection history (unpublished data).

Although the biology and genetics of drug resistance to each component drug of ACT has been studied separately, less is known about the factors influencing ACT resistance and its acquisition, as a whole. For instance, assuming that two independent mutations are required to confer resistance to the two component drugs, it is not known whether the mutation rate in *Plasmodium* spp. can produce the two mutations in one parasite (in one host) at a sufficient frequency, or whether these mutations confer fitness advantages if the two component drugs are administered at the same time.

In order to address the above issues, we have used *Plasmodium chabaudi* to investigate the evolution of resistance to ACTs, by selecting *in vivo* resistance to ATN + MF through prolonged exposure to both drugs simultaneously. Resistant parasites were successfully generated, and their *atpase6*, *ubp-1* and *mdr1* genes were compared to those of the sensitive progenitor.

## Results

### Selection and cloning of artesunate + mefloquine- resistant parasites

AS-ATN (Fig. 1), a parasite clone previously selected for limited artesunate (ATN) resistance [28], was used as the starting biological sample and subjected to prolonged and increasing exposure to the two drugs over many generations.

**Figure 2:**
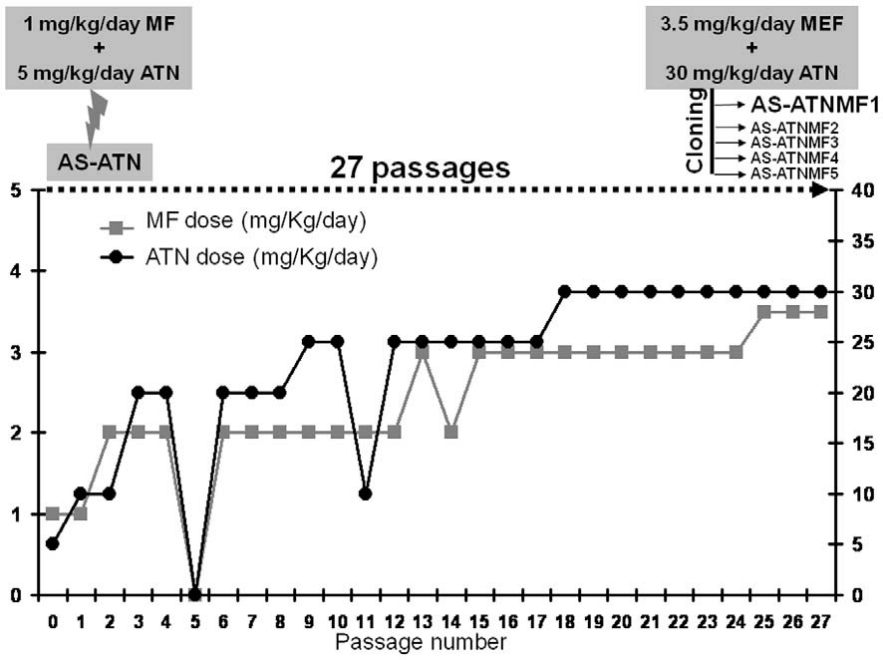
*In vivo* evolution of resistance to artesunate + mefloquine. Artesunate (ATN) and mefloquine (MF) were given together to *P. chabaudi*-infected mice over many generations. After twenty seven sub-inoculations under increasing ATN + MF exposure, the drug-resistant population was cloned. One of these clones, denoted AS-ATNMF1, was selected from subsequent studies.

The AS-ATN parasite was first inoculated into a group of mice which received a sub-curative drug dose consisting of 5 mg/kg of ATN +1 mg/kg of mefloquine (MF), given over a 3-day course (Figure 2). After approximately seven days, parasites which survived this treatment were sub-inoculated into uninfected mice and treated with a slightly higher dose. This procedure was repeated using drug doses which were adjusted at each stage, depending on the extent of parasite recovery during the previous treatment (Figure 2). After twenty-seven of such passages, the drug-selected parasites were shown to tolerate 30 mg/kg/day ATN +3.5 mg/kg/day MF. This line is likely to contain parasites with different genotypes and phenotypes. Therefore, they were subjected to a cloning process in order to isolate and propagate a genetically homogeneous ATN + MF-resistant parasite. Five clones were obtained from a group of 20 mice each receiving a calculated inoculum of 0.5 parasites/mouse in (Figure 2) and eight clones from a group of 20 mice injected with a mean of 1 parasite/mouse (data not shown). The fastest growing clone within the first group, termed herein AS-ATNMF1 (Figure 2), was chosen for subsequent phenotypic and genotypic evaluation.

### Evaluation of ATN + MF resistance in drug tests

The AS-ATNMF1 clone described above was submitted to drug-treatment in parallel with parasites of the AS lineage which had never been exposed to ATN + MF. These controls comprised AS-3CQ (sensitive to both ATN and MF), the MF-resistant parasite AS-15MF, and the AS-ATN progenitor. ATN + MF responses were thus evaluated under 40 mg/kg/day ATN +4 mg/kg/day MF administered for 3 days in groups of five mice along with untreated controls. The above doses were slightly higher than those used at the end of the selection procedure because parasites were growing well under 30 mg/kg/day ATN +3.5 mg/kg/day MF (data not shown).

**Figure 3:**
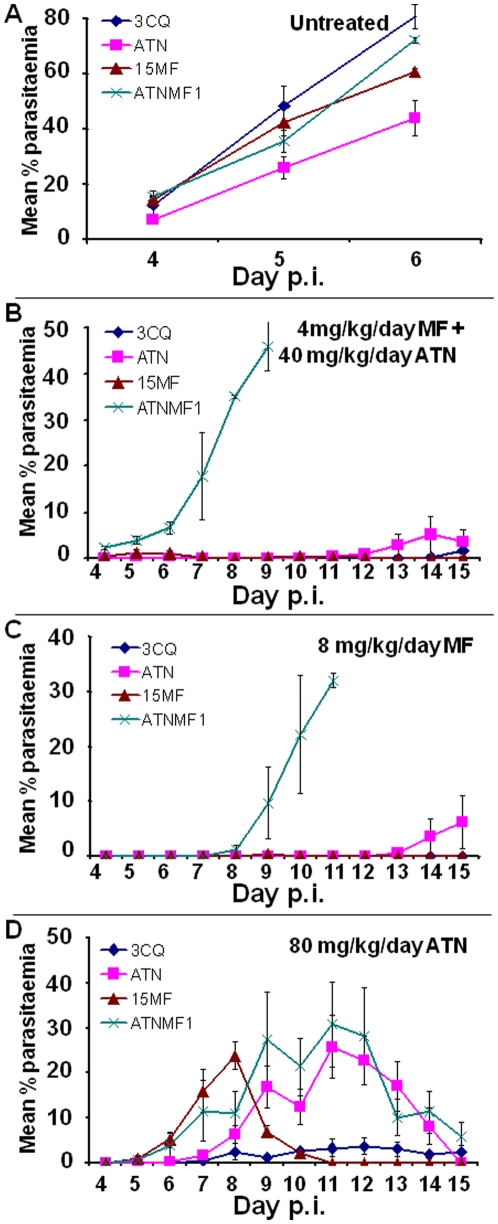
Drug test results. Each line represents the evolution of parasitaemia in each parasite clone from day 4 post-inoculum (p.i) in the absence of treatment (a), under artesunate + meloquine treatment (b), under mefloquine treatment (c) or under artesunate treatment (d). Each data point is a mean of % parasitaemias ± SE resulting from reads of groups of two and five mice in the untreated and treated groups, respectively.

In the absence of treatment, all four parasite clones grew well in mice, as expected, with peak parasitaemias reaching between *circa* 40% (AS-ATN) and 80% (AS-ATNMF-1) between days 5 and 6 post-inoculum (p.i.) (Figure 3A). Under ATN + MF treatment, no parasites were detected in mice infected with either AS-3CQ or AS-15MF over a 15-day p.i. follow-up, whilst AS-ATN showed a slight (<5%) recrudescent parasitaemia (days 12–15) (Figure 3B). Thus, AS-3CQ, AS-15MF and AS-ATN were deemed as sensitive to ATN + MF combination treatment. In contrast, mice infected with the drug-selected clone AS-ATNMF1 failed to clear parasitaemias after drug-treatment, with parasites being first detected on day 8 p.i. and peak parasitaemias of *circa* 30% occurring on day 11 p.i (Figure 3B).

We conclude that an ATN + MF-resistant parasite was successfully selected and cloned. The resistance phenotype was stable after successive freeze-thaw cycles (data not shown).

### Evaluation of response to individual drugs (ATN and MF)

In order to investigate whether the evolution of resistance to ATN + MF treatment also increased the resistance to the individual components of the ACT, we evaluated the individual ATN and MF responses of AS-3CQ, AS-ATN, AS-15MF and AS-ATNMF1. Drug doses used in these tests were higher for individual drugs than those used in the combination treatments because individual components are generally less effective when used as monotherapy.

In both ATN and MF tests, all untreated control parasites grew as expected, with parasitaemias reappearing in infected mice on day 4 p.i. and reaching peak values one to two days later (Figure 3A).

The MF-resistant phenotype of AS-ATNMF1 was very marked in comparison to the remaining clones (Figure 3C). Thus, under a daily dose of 8 mg/kg/day, AS-ATNMF1 parasites recrudesced on day 4 p.i. and reached a high peak parasitaemia of 46% on day 9 p.i. In contrast, AS-3CQ was classified as MF-sensitive, because parasites were not detectable throughout the course of the experiment (Figure 3C). Interestingly, AS-15MF was previously defined as MF resistant at 6 mg/kg/day [27] but here it is sensitive to 8 mg/kg/day (Figure 3C). Finally AS-ATN showed a small peak parasitaemia which was delayed until at least day 15 p.i (Figure 3C). Therefore, AS-ATNMF1 has increased MF resistance relative to its progenitors and to the independently selected MF-resistant clone AS-15MF.

In the ATN response test, a dose of 80 mg/kg/day (3 day treatment) was used. For the ATN- and MF-sensitive parasite AS-3CQ, recrudescence was first observed on day 13 p.i., with peak parasitaemias reaching 9% on day 14 (Figure 3D). In contrast, AS-ATNMF1 parasites appeared on day 5 p.i. and reached 28% on day 9 p.i (Figure 3D). The MF-resistant parasite, AS-15MF, also displayed some resistance to ATN, first producing observable parasites on day 4 p.i. and peak parasitaemias of 24% on day 8 p.i (Figure 3D). The parasitaemia cleared rapidly. ATN-resistant AS-ATN parasites showed an intermediate phenotypes, producing parasites on day 6 p.i peak parasitaemia of 28% on day 11 p.i (Figure 3D). The ATN drug tests therefore show that AS-ATNMF1 does have increased resistance to ATN relative to its progenitor, AS-ATN.

AS-ATNMF1 therefore shows increased resistance to both MF and ATN components individually, as well as to their combination ATN + MF.

### *atpase6*, *ubp-1* and *mdr1* genotypes in AS-ATNMF1

*P. chabaudi* AS-ATNMF1 parasites were interrogated for mutations in candidate genes previously implicated in resistance to each of the drugs employed here. Thus, we first searched for the presence of point mutations in those genes proposed to be involved in the increased tolerance to ART derivatives, namely *atpase6* [10], [12] and *ubp1* [29]. To this purpose, the whole coding sequence of both genes was sequenced and no differences were found between AS-ATNMF1 and its progenitor AS-ATN.

Changes in the copy number of the *pfmdr1* gene are frequently found to be present in MF-resistant parasites [16], [27] and have also been reported to confer selective advantage in the presence of ART drug pressure [16], [30]. In this context, we investigated by real time PCR whether the evolution of ATN + MF-resistance had selected parasites with increased *mdr1* copy number or mRNA expression. Because both AS-ATN and AS-3CQ are known to harbour a single copy of the *mdr1* gene [28], [31], and AS-15MF has been shown previously to carry two copies [27], these clones were also analysed. Real-time qPCR was thus carried out using a TaqMan® assay as detailed in the “Materials and Methods” and data were expressed as the mean N-fold difference ± SE in *mdr1* gene copies of each of the above parasite clones in relation to either AS-SENS or AS-ATN.

**Figure 4:**
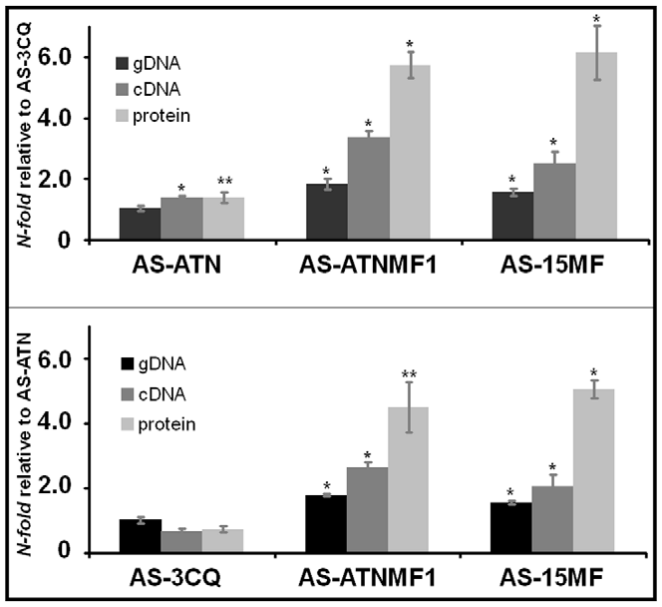
Differences in *mdr1* genomic DNA, cDNA and protein amounts between the different parasite clones. Columns represent mean of five independent experiments with corresponding Standard Error bars. Cases were differences in relative *mdr1* amounts between a particular parasite and its one copy comparator (AS-3CQ or AS-ATN) are statistically significant after Student's t-tests are represented by * (p≤0.01) or ** (0.05≥p≥0.01).

AS-ATNMF1 displayed 1.8±0.09 and 1.8±0.37 *mdr1* copies relative to the single-copy AS-ATN progenitor and AS-SENS, respectively (Figure 4). Conversely, the estimated copy number between AS-ATNMF1 and the two-*mdr1* copy parasite AS-15MF was approximately equal (Figure 4). These results provide strong indication that AS-ATNMF1 carries two copies of the *mdr1* gene, one of which was gained in the course of the evolution of resistance to the ATN + MF combination treatment.

In line with an increase in *mdr1* copy number, mRNA levels were also found to be increased in AS-ATNMF1, showing that the genomic amplification is accompanied by increased transcription (Figure 4). Thus, the fold-increase of AS-ATNMF1 *mdr1* mRNA transcripts was 2.6±0.17 and 3.4±0.22, relative to its single copy number progenitor AS-ATN and to AS-3CQ, respectively. Interestingly, relative mRNA levels in both AS-ATNMF1 and AS-15MF are higher than those of gene copy number, as determined by cDNA/genomic DNA ratios (Table 1).

**Table 1:** Relationships between genomic DNA, transcript and protein amounts of *mdr1* between the different parasite clones.

Expression ratios				
AS-3CQ as control	AS-3CQ	AS-ATN	AS-15MF	AS-ATNMF1
cDNA/gDNA	-	1.40	1.56	1.88
Protein/cDNA	-	1.00	2.48	1.64
Protein/gDNA	-	1.40	3.87	3.11

AS-ATN as control				
cDNA/gDNA	0.70	-	1.4	1.44
Protein/cDNA	1.00	-	2.4	1.70
Protein/gDNA	1.00	-	3.33	2.50

Values were determined by dividing the mean N-fold expression between each molecular species.

### MDR1 protein levels are increased in AS-ATNMF1

In order to determine whether the increase in genomic copies and transcription of the *mdr1* gene was accompanied by an increase in its product, we measured the relative amount of MDR1 protein in the different parasite clones, using quantitative Western blot analysis.

**Figure 5:**
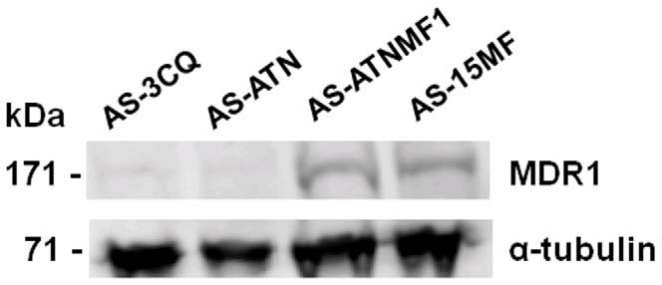
Typical Western blot result depicting MDR1 expression in the artesunate + mefloquine resistant clone AS-ATNMF1. Hybridization was carried out with α anti Pgh1 antibodies and α anti tubulin antibodies. AS-3CQ and AS-ATN were used as one-*mdr1* copy controls and AS-15MF was used as the two-*mdr1* copies control.

Because MDR1 levels have been shown to be expressed in correlation with the gene copy number in previous studies [20], [30], we expected protein amounts to be approximately doubled in AS-15MF and AS-ATNMF1, in line with the quantitative gDNA and mRNA analysis. We observed a considerable increase in the amount of MDR1 in both the MF-resistant parasites, AS-15MF and in AS-ATNMF1 in relation to the *mdr1* single copy parasites AS-3CQ and AS-ATN (see Figure 5). Thus, AS-15MF displayed a 6.2±0.88 and 5.0±0.30 fold increase in MDR1 levels in comparison to AS-3CQ and AS-ATN, respectively (Figure 4). For AS-ATNMF1, the amount of MDR1 was augmented 5.6±0.44 fold when compared to AS-3CQ, and 4.5±0.80 fold in relation to AS-ATN (Figure 4). There is thus a disproportionate increase in the expression of MDR1 protein relative to gene copy number in the resistant parasites (Table 1). These observations suggest that the post-translational regulation of MDR1 may be complex.

Finally, we compared MDR1 levels between the single *mdr1* copy ATN-resistant parasites AS-ATN and AS-3CQ. Interestingly, there was a small but significant increase of MDR1 protein in AS-ATN [1.4±0.0.17 (p = 0.05)] relative to AS-3CQ (Figure 4).

## Discussion

Here, we aimed to generate a parasite resistant to the artesunate + mefloquine (ATN + MF) version of ACT. For this, AS-ATN, a parasite clone previously selected for limited artesunate (ATN) resistance [28], was used as the starting biological sample and subjected to prolonged and increasing exposure to the two drugs over many generations. The reasons for using this parasite were two-fold: firstly, the possibility that *mdr1* would be amplified following ATN + MF pressure could be investigated, because AS-ATN harbours a single *mdr1* copy. Secondly, since ATN-resistant parasites have recently been reported from South-East Asia [7]–[9], where the ATN + MF combination is widely deployed, the present results may provide important early insights on the evolution of ATN + MF resistance on an ATN-resistant background.

Two major key outcomes of this work deserve particular consideration. First, by deriving an ATN + MF-resistant clone, we have shown that resistance may evolve to two drugs and their combination even when the two drugs are given simultaneously. Consequently, the chance of selecting a resistant parasite harbouring mutation(s) that enable it to withstand the effects of both drugs within a combination may be higher than previously perceived. That strategy is predicated on the principle that each of the drugs should have different modes of action and thus elicit distinct parasite response mechanisms [2]. There may be evolutionary implications to these results. One is that because this is an artificial *in vivo* model where drugs are given initially at low doses and slowly increased over a number of generations in a controlled environment, there is a higher chance of resistant mutants surviving onto subsequent generations. Unfortunately, however, non-optimal drug dosing is also often encountered in natural parasite populations of *P. falciparum* because of different factors, such as inadequate compliance to treatment [4], rapid drug clearance in some patients [32] or the distribution of sub-standard drug formulations that contain lower amounts of the active compounds [33]. Therefore, a similar scenario to that modelled here may also occur in human malaria, promoting selection of ACT-resistant mutants in a similar fashion.

It is also possible that the rate of occurrence of multiple mutations conferring resistance to the drug combination is higher than expected and/or that both drugs actually share common mechanisms of resistance. We note that the sensitive progenitor parasite AS-ATN used here already has a lower level of resistance to ATN and carried a pre-existing mutation in the *ubp-1* gene [29] which may have facilitated further evolution of resistance. However, because *P. falciparum* parasites with a markedly reduced susceptibility to ATN have recently been reported from South-East Asia [7]–[9], the ATN-resistant background used here may help us to gain insights into the further evolution of resistance to ATN + MF in that field situation.

The second important consequence of the present work is that it demonstrates the selection of parasites harbouring two copies of the *mdr1* gene by *in vivo* ACT treatment. This was the only genetic alteration detected following inspection of *ubp1*, *atpase6* and *mdr1* itself, the major candidate genes previously reported to be associated to resistance to the drugs used here. Interestingly, the pre-existing V2697F mutation in the *ubp-1* gene was retained following drug selection and no further mutations arose in this gene. Is it possible that the *ubp-1* mutation is acting in concert with the *mdr1* duplication in determining the ATN + MF resistance phenotype by, for instance, reducing ubiquitination of MDR1 and either allowing its increased turnover or modifying its localisation within the parasite? If so, we might have expected that the mefloquine (MF)-resistant clone AS-15MF would also be resistant to the ATN + MF combination because it too bears a mutation in *ubp-1* as well as a duplication of the *mdr1* gene. However, whilst this genotype is sufficient to render it resistant to both MF (at a lower level than AS-ATNMF1) and ATN when these drugs are given separately, our data shows that it is not resistant to both drugs simultaneously. In any case, AS-ATN also carries an *ubp-1* mutation and is also fully sensitive to ATN + MF. The acquisition of an extra *mdr1* copy in AS-ATN is accompanied by the acquisition of resistance to ATN + MF. In summary, whilst AS-15MF and AS-ATNMF1 share both a mutation in *ubp-1* and two *mdr1* copies, only the latter is resistant to the ATN + MF combination. Therefore, these data appear to indicate that whilst both genes may be involved in resistance to these drugs, the ATN + MF resistance phenotype is more complex and is likely to involve mutation(s) in other gene(s). Further genomic, genetic and functional analyses will be required to establish whether other mutations are involved in ATN + MF resistance in AS-ATNMF1.

Although it is uncertain whether selection of the *mdr1* duplication and its consequent overexpression was driven by ATN, MF, or both, this result is similar to a number previous studies suggesting a role for this gene in resistance to aminoquinolines and artemisinin derivatives [19], [21], [22], [24], [30], [34]. However, we suggest that repeated MF treatment alone might select parasites with *mdr1* duplications and that these parasites would show (as in this study) increases in MF-resistance, resistance to the combination and, perhaps, slight increases in ATN resistance. The present work is therefore providing further support for the participation of *mdr1* in a true multi-drug-resistant phenotype for artemisinins and their partner drugs. Additionally, it is suggested here that even under the putative scenario of MF being the major selection pressure for *mdr1* amplification, the presence of an ART partner drug is not antagonistic to this process. As a matter of fact, the opposite is more likely to occur as suggested here, and also supported by previous data where disruption of one of two *mdr1* copies in *P. falciparum* FCB has shown to result in a two-fold increase in ART susceptibility [22]. Collectively, these observations have major public health consequences, as a shared drug defence mechanism will undermine the efficacy of Artemisinin Combination Therapies (ACTs) as a whole, and facilitate the evolution of resistance.

Interestingly, the *mdr1* and MDR1 expression data suggest that changes in *mdr1* copy number may be accompanied by larger changes in the expression of both mRNA and protein. This difference was observed exclusively in the clones that harboured pre-existing *ubp-1* mutations (AS-ATN, AS-15MF and AS-ATNMF1), but not in AS-3CQ which carries a wild-type allele. This unexpected result contradicts a simple model of post-transcriptional or post-translational regulation of MDR1 and was not observed previously in *P. falciparum* parasites selected *in vitro* for MF and ART resistance [20], [30]. One possible explanation could be that *ubp1* regulates MDR1 ubiquitination. Either the 26 S proteasome-dependent turn-over of MDR1 is modified from one cellular location preferentially, or the distribution or trafficking of MDR1 within the cell is modified. Either of these outcomes could perturb the overall regulation of *mdr1* mRNA and protein expression. Although this hypothesis is preliminary and highly speculative, the stability and steady-state expression of the human homologue Pgp-1, has been shown to be amenable to ubiquitin-dependent regulation in drug-resistant cancer cells [35], providing an experimental precedent for the present observations.

In the absence of fully effective antimalarial treatments, the evolution of resistance to ACTs would represent a major shortcoming in worldwide efforts to control *Plasmodium falciparum* malaria. Unfortunately, recent global concerns about the future efficacy of ACTs have been triggered by the first indications that artemisinin's efficacy may be waning [7]–[9]. Because we lack new effective drugs and effective treatments, there is a pressing requirement to understand how the efficacy of present ACT treatments may be protected. The results reported here may contribute to improved understanding of the biology, dynamics and genetic traits governing ACT resistance, which will provide evidence-based information for improved drug surveillance and future choice of the ACT combination components.

## Materials and Methods

### Ethics Statement

All animal work was conducted according to relevant national and international guidelines after approval by the Ethics Committee of the Instituto de Higiene e Medicina Tropical of Lisbon, Portugal, under PARECER 2/2006 from August 1^st^ 2006.

### Parasites, hosts and strategy for selecting ACT-resistant parasites

Cloned parasite lines from the isogenic AS lineage of the rodent malaria parasite *Plasmodium chabaudi* were used in this work. Each of these had been previously characterized for its response to the drugs under study (Figure 1) and their genotype had been assessed for each of the three candidate genes *atpase6*, *ubp-1* and *mdr1* (Figure 1). Our objective was to generate resistance to the artesunate (ATN) + mefloquine (MF) version of ACT. The strategy to achieve resistant parasites was based on the prolonged exposure of parasites to consecutive small increments in drug doses in treated mice over many generations. To achieve this goal, parasites resistant to a low level of ATN (*P. chabaudi* AS-ATN) were exposed to a combination of ATN and MF. Thus, blood containing the parental clone AS-ATN was thawed and inoculated intraperitoneally into two CD-1 6–8 week-old mice. When these mice reached peak parasitaemia, donor blood was extracted to inoculate 10exp7 parasitized red blood cells (pRBC), diluted in citrate saline, into two groups of two mice each. One group was left untreated and parasites were sub-inoculated every seven days into two uninfected mice in the absence of treatment and in parallel with the treated group. The remaining two mice were treated with a combination of ATN and MF dissolved on DMSO on days 3, 4 and 5 post-inoculum (p.i.) by gavage. Infection progression in individual mice was monitored from day 3 onwards by counting the percentage parasitaemia in Giemsa-stained thin blood smears. The treated mouse containing the highest parasitaemia on day 7 p.i., was then exsanguinated by section of the brachial artery under general anesthesia and served as donor for the next round of drug selection. The starting drug-selecting dose was of 1 mg/Kg/day of MF administered together with 5 mg/kg/day of ATN and it was increased whenever parasitaemias reached above 2% on day 7, under drug treatment. After 27 rounds of drug selection, parasites under drug pressure were cloned by limiting dilution [36]. The parasite population surviving treatment was cloned by limiting dilution at the end of this period.

ATN was kindly donated by Dafra Pharma International™. Mefloquine was purchased from Roche™. Drugs were freshly diluted into DMSO (Merck™) prior to administration.

### Drug sensitivity test

In order to assess the success of drug resistance evolution, parasites were tested for their drug responses, at the end of the drug selection period, as follows. In these tests, 4–6 week-old inbred Balb/c mice were used. For each drug to be tested (artesunate, mefloquine or artesunate + mefloquine) mice were divided into four groups of 10 mice and each mouse within the group was inoculated with 10exp7 pRBC with one of the following parasite clones (Figure 1): i) the drug-selected clone AS-ANTMF1, ii) its sensitive progenitor AS-ATN, resistant to low levels of ATN), iii) a MF resistant clone denoted AS-15MF, and iv) AS-3CQ, which is resistant to low doses of chloroquine, but sensitive to both MF and ATN. Each group was further divided into two sub-groups, one of which was given the diluting vehicle DMSO and the other was treated with the drug being assayed. Treatment regimes consisted of a daily dose first administered on day one p.i. for three consecutive days. Individual percentage (%) parasitaemias were then followed from day 4 onwards and up to day 15 and results were expressed as daily average % parasitaemias from the five mice within each experimental group. In order to obviate suffering, mice were sacrificed through cervical dislocation whenever they reached peak parasitaemias.

### PCR and sequencing

Blood from mice bearing each of the parasites mentioned above was dried in Whatman n°4 filter paper for DNA extraction. 1 cm^2^ of the filter paper was incubated overnight in 1 ml PBS (Sigma) +0.5% saponin (Sigma) at 4°C. The solution was removed and the filter paper was incubated with 1 ml PBS at 4°C for 30 minutes. After the removal of PBS, the filter paper was incubated at 100°C with pre-heated 200 µl PBS +5% Chelex-100 (BioRad™) for 10 minutes and vortexed vigorously every 5 minutes. The tubes were centrifuged at 14000 rpm for 2 minutes and the supernatant was placed into a fresh tube. The supernatant (containing the DNA) was centrifuged again in order to insure the complete removal of the Chelex-100 matrix and stored at −20°C.

The sequences of the *P.faciparum* ortholog genes *pcatpase6* (PCAS_020540) and *pcubp-1* (PCAS_020720) were obtained from plasmoDB and used for comparing the fragments obtained when analysing DNA from the parasites selected here with their progenitors. The primer sequences as well as the cycling conditions for the *pcatpase6* gene were described previously by Afonso et al, 2006 [28] whereas the primer sequences and cycling conditions for the gene *pcubp1* were described previously by Hunt et al, 2007. The only exception are the primers surrounding the V2697F mutation, characteristic of the AS-ATN clone [29]. The forward sequence is 5′-GTTACCAATTGATACGACTG-3′ and the reverse sequence is 5′CAGAATTAGTATGAGGTGGC-3′. 1 µl of DNA from each sample was used as template in 50 µl reactions. The other reagents were added to a final concentration of 1,5 mM MgCl2, 0.2 mM dNTP (each), 0.2 pmol/µl forward and reverse primers, and 1.25 U of Go Taq Flexi DNA Polymerase (Promega). The cycling conditions were: denaturation at 94°C for 3′, 15 cycles of 94°C for 15′′, 40°C for 45′′ and 68°C for 4′, followed by 30 cycles of 94°C for 1′, 45°C for 45′′ and 72°C for 3′. The final elongation was run for 10′ at 72°C.

PCR products were sequenced by de-deoxy sequencing and sequences were aligned using Multi-Align [37], a multiple sequence alignment software available online (http://bioinfo.genotoul.fr/multalin/multalin.html).

### Assessement of *pcmdr1* copy number and mRNA expression by quantitative Real-time PCR

DNA samples above were used to determine the copy number of the *pcmdr1* (PCAS_123820) in ATN + MF-resistant clones, using a TaqMan™ probe assay. Quantification was made based on relative estimates of genomic target DNA amounts between different parasite clones, using the 2^−ΔΔct^ method [38], and data was normalized against the house keeping gene *pc-αtubulin* ( PCAS_052240). Parasite clones of known *mdr1* copy number, AS-SENS, AS-ATN and AS-15MF, were used as controls, the two former harbouring one copy and the latter, two copies of *mdr1* [27]–[28].

Oligonucleotide primers and TaqMan™ probes (Table 2) were obtained through the services of TIBMOLBIOL and designed such as PCR reactions for both genes could be ran in the same plate (using the same cycling conditions). DNA samples, primers and probes were added to Faststart Universal Probe Master Mix with ROX (Roche™), according to the manufacturer's instructions and submitted to 95°C for 10 minutes followed by 50 cycles of denaturation at 95°C for 15 seconds and annealing/extension at 57°C for 1 minute.

**Table 2:** Oligonucleotide primers and TaqMan probes used to ascertain *mdr1* copy number and transcription levels.

*P. chabaudi mdr1*			
Designation	Sequence	Position in gene	*TM* (°C)
*Pcmdr1-F*	CACACAATTTGAAAGACGTTGACT	1530–1553	56.9
*Pcmdr1-R*	ATTTAATGAAGAATCGCTACTTCCG	1714–1690	57.2
*Pcmdr1-Tp*	FAM-TCATGGTGAATCCATTTTCCATTGCTTCT-BBQ	1688–1660	65.6

	*P. chabaudi tubulin*		
Designation	Sequence	Position in gene	*TM* (°C)
*Pctub-F*	TGCTCCAATTATTAGTGCTGAAAAAG	318–343	58.1
*Pctub-R*	ACAGCAAGCCATATATTTTCCATGTC	450–425	59.1
*Pctub-Tp*	FAM-CCTGCATCTATGATGGCAAAATGTGATCCT-BBQ	394–423	66.8

*F*: forward; *R*: Reverse; *Tp*: TaqMan probe.

In order to evaluate relative differences in *mdr1* expression at the mRNA level, the same procedure as that described above was applied to template cDNA of each of the parasite clones. For this, RNA was first extracted from blood stage parasites in order serve as template for cDNA synthesis as follows. Parasite-infected mice were bled out and the mixture of blood and Citrate Saline Solution was filtered in a cellulose (CF11, Sigma) 5 ml column. This was then centrifuged at 3000 rpm for 5 minutes at room temperature and the supernatant was discarded. The volume of the erythrocyte pellet was estimated and an equal volume of Citrate Saline Solution was added to it and mixed gently. RNA from this pellet was then extracted using RNA Blood Kit 100 (BioRad™) following the manufacturer's instructions with minor changes described bellow. Briefly, 300 µl of blood was mixed by inversion with 900 µl of RBC Lysis Solution, provided with the kit. The mixture was incubated at room temperature for 10 minutes and then centrifuged at 14000 rpm for 10 minutes at 4°C. The supernatant was discarded leaving approximately 20 µl of residual liquid that was thoroughly mixed with the pellet. Subsequently, 300 µl of RNA Lysis Solution was added and mixed by pipetting repeatedly. 100 µl of the Protein-DNA Precipitation Solution was added and the mixture was incubated on ice for 10 minutes. The samples were centrifuged at 14000 rpm for 5 minutes at 4°C and the pellet was discarded. 300 µl of 100% Isopropanol was added to the supernatant and centrifuged at 14000 rpm for 5 minutes at 4°C. The supernatant was poured out and the tube was briefly dried using an absorbent paper. The pellet was washed in 70% Ethanol. After being centrifuged again under the same conditions as above, the supernatant was rejected and the tube was allowed to air-dry for 15 minutes. 50 µl of RNA Hydration Solution was added to the dry pellet and the tube was kept on ice for 30 minutes.

The RNA sample obtained as described above was either used immediately for cDNA synthesis or kept at -70°C. Before starting cDNA synthesis, samples were treated with DNAse to ensure the removal of any contaminant DNA. Thus, 4 µl of the RNA sample was mixed with 1.0 µl of 10× DNAse Buffer (FERMENTAS™), 1.0 µl of 1 U/µl DNAse (FERMENTAS™) and 5.0 µl of DEPC treated water and incubated for 15 minutes at room temperature. 1.0 µl of 25 mM EDTA was added to the reaction mixture and incubated at 65°C for 5 minutes. Reverse transcription of the DNAse treated RNA began by the addition of 2 µl of 10× concentrated Hexanucleotide Mix (Roche™). The tube was placed in a dry bath at 70°C for 5 minutes and later cooled on ice. After that, a mixture consisting of 4 µl of 10× M-Mulv Buffer (FERMENTAS™), 2 µl of deoxynucleoside triphosphate mix (10 mM each), 3 µl of 100 mM DTT, 0.5 µl of 400 U/µl RNA inhibitor Ribolock™ (FERMENTAS™), and 7.5 µl of DEPC treated water was prepared. 17 µl was added to the mixture of DNAse treated RNA and Hexanucleotides and it was incubated at 25°C for 5 minutes. 1 µl of 200 U/µl M-MuLV Reverse Transcriptase (FERMENTAS™) was added and the reverse transcription was performed by one cycle of 25°C for 10 minutes, 42°C for 1 hour, and 70°C for 10 minutes. Samples were allowed to cool on ice and kept at −20°C prior to use.

### Western blots

To prepare protein extracts were prepared from erythrocyte stage parasites, mice were injected with infected blood and exsanguinated upon reaching peak parasitaemia. Collected blood was diluted in Citrate Saline Solution and then filtered once using a cellulose (CF11, Sigma) 5 ml column and centrifuged at 3000 rpm for 5 minutes. The pellet was washed twice in ice-cold PBS and re-suspended into two volumes of PBS. The mixture was divided into 500 µl aliquots. RBC lysis was performed by adding 1 ml of Hypotonic Ammonium Chloride Solution and incubating at 4°C for 3 minutes. The samples were centrifuged at 10000 rpm for 7 minutes at 4°C and the supernatant was discarded. The pellet was washed twice in PBS and finally centrifuged at 10000 rpm at 4°C for 7 minutes. Pellet volume was estimated and re-suspended into twice the volume of Loading Buffer. Samples were kept at −20°C, and prepared as follows upon usage: protein amounts were determined by Bradford assay (BioRad™, Germany). Samples were then run on NuPAGE Novex Tris-Acetate gels (Invitrogen, Germany) and transferred onto a 0.2 µm PDVF membrane (BioRad™, Germany). Membranes were blocked overnight at 4°C using 5% milk in PBS. Primary antibodies (α-Pgh-1 and α-tubulin, diluted 1∶1,000 and 1∶2,000, respectively) were incubated for 1 h at room temperature in 1% BSA+PBS. Membranes were washed three times using PBS+0.1% Tween for 10 min at room temperature and then blocked again in 5% milk in PBS for 1 h. Secondary antibodies (Alexa Fluor 680 goat anti-rabbit IgG, or Alexa Fluor goat anti-mouse IgG, both diluted 1∶10,000) were added to 1% BSA/PBS for 30 min at room temperature. After washing four times in PBS +0.1% Tween for 5 min, signals were read using an Odyssey-Li-cor infrared imaging system (Li-cor Biosciences). Fluorescence intensities for MDR1 (α -Pgh-1) were normalized using fluorescence intensities measured for α -tubulin. The resulting values were then expressed in relation to AS-3CQ or AS-ATN.

The results were then expressed as means of five replicates and a Student's t-test was used to determine statistical significance of results obtained.
